# Effectiveness of Different Exercise-Based Interventions Combined or Not with Electrotherapy Versus McKenzie Method Alone for Nonspecific Chronic Neck Pain: A Systematic Review and Meta-Analysis

**DOI:** 10.3390/jcm15051689

**Published:** 2026-02-24

**Authors:** Cristian Sánchez-Ferre, Inmaculada Carmen Lara-Palomo, Ana Belén González-Nula, José Abad-Querol, Silvia Gómez-García, Elena Álvarez-López, Guillermo Adolfo Matarán-Peñarrocha, Adelaida María Castro-Sánchez

**Affiliations:** 1 Department of Nursing, Physiotherapy and Medicine, University of Almeria, 04120 Almeria, Spain; csf088@inlumine.ual.es (C.S.-F.); ilp813@ual.es (I.C.L.-P.); agn030@inlumine.ual.es (A.B.G.-N.); 2 Department of Physiotherapy, Huercal de Almeria Centre, Distrito Sanitario Almeria, Andalusian Health Service, 04230 Almeria, Spain; jose.abad.querol.sspa@juntadeandalucia.es; 3 Department of Physical Medicine and Rehabilitation, Hospital Universitario Torrecárdenas, Andalusian Health Service, 04009 Almeria, Spain; silviagomezgarcia@hotmail.com (S.G.-G.); elena.alvarez19@gmail.com (E.Á.-L.); 4 Family Medicine and Primary Care, Distrito Sanitario Málaga, Andalusian Health Service, 29009 Málaga, Spain; guillelemur@gmail.com

**Keywords:** nonspecific neck pain, chronic pain, exercise therapy, electrotherapy, transcutaneous electrical nerve stimulation, McKenzie method, systematic review, meta-analysis

## Abstract

**Background/Objectives:** Chronic nonspecific neck pain is a common global health problem that diminishes people’s quality of life and functionality. Strengthening and mobility exercises are a fundamental tool in managing this condition. Combined treatment with electrotherapy appears to have promising results; however, the evidence is limited. The aim of this review was to compare the effectiveness of therapeutic exercise combined with electrotherapy versus the McKenzie method alone in improving pain and disability in adults. **Methods:** A systematic review and meta-analysis of randomized clinical trials was conducted following the PRISMA guidelines. Studies published up to June 2025 were extracted from major scientific databases. High-quality studies evaluating therapeutic exercise with or without electrostimulation and studies evaluating the McKenzie method alone were analyzed, measuring short-term pain and disability through meta-analysis using random-effects models. The risk of bias of the included studies was assessed using the Cochrane Collaboration tool. **Results:** Seven studies were included (*N* = 441). The combination of therapeutic exercise with electrotherapy showed a significant reduction in pain (SMD −0.76 [−1.36, −0.16] (*p* = 0.01; 95% CI)), without additional benefits for disability (SMD −0.94 [−2.08, 0.20] (*p* = 0.1; 95% CI)) compared to exercise alone. Similarly, the McKenzie method presented statistically significant differences compared to other active interventions in reducing pain (SMD −0.61 [−1.01, −0.21] (*p* = 0.003; 95% CI)), while no significant differences were found for disability (SMD −0.31 [−1.78, 1.15] (*p* = 0.67; 95% CI)). Heterogeneity among studies was generally high. The results show short-term effects measured after completion of the intervention. **Conclusions:** Electrotherapy combined with exercise may provide short-term relief of nonspecific chronic neck pain, although the certainty of evidence is very low. Interferential current plus exercise and isolated McKenzie exercises showed short-term pain improvements, with no consistent benefits for disability. The methodological limitations, heterogeneity, small samples, and short follow-up warrant cautious interpretation. High-quality trials with standardized protocols and longer follow-up are needed.

## 1. Introduction

Nonspecific neck pain (NSNP) is a common condition that can affect a large portion of the world’s population at some point in their lives, causing significant discomfort and, in many cases, impacting quality of life and functionality [1]. This condition may manifest in restrictions in cervical and upper thoracic movement, headaches, and pain radiating to the upper limbs, highlighting its impact on daily and work activities [2]. Unlike other types of neck pain associated with specific pathologies, this type of pain does not present an identifiable pathognomonic cause through clinical or imaging studies, which makes its management challenging and underscores the importance of a comprehensive assessment that considers physical and psychosocial aspects [1,3].

Neck pain represents a substantial clinical and socioeconomic burden worldwide, contributing significantly to disability and healthcare utilization; however, beyond its prevalence, the main clinical challenge lies in identifying effective, evidence-based therapeutic strategies capable of producing sustained improvements in pain and function [4]. It is important to consider risk factors to address the growing challenge posed by neck pain [3,4]. Some of these factors are mechanical, such as poor posture or repetitive movements; however, stress and other psychological components contribute to the chronicity of disability and discomfort [1]. It is also crucial to consider other risk factors that predispose to chronicity, such as age (over 40 years), associated low-back pain, history of cervical discomfort, frequent cycling, decreased hand strength, and pain-related anxiety [1,2,3].

The therapeutic approach to NSNP is based on an integrated strategy that combines symptom management with active interventions, such as muscle strengthening, joint mobility exercises, and vestibular rehabilitation, if needed, in addition to strategies that promote psychological well-being [1,5]. Nevertheless, despite the widespread use of exercise-based interventions, there is considerable heterogeneity in treatment protocols, dosage, progression strategies, and reported clinical outcomes, resulting in uncertainty regarding the most effective therapeutic approach. In terms of prevention, it is essential to adopt proper postural habits and maintain an exercise routine that encourages flexibility and muscle strengthening; a multidisciplinary approach is crucial to provide effective treatments based on the best available evidence [1,3].

The McKenzie method, also known as Mechanical Diagnosis and Therapy (MDT), is a widely used tool designed to assess and treat musculoskeletal disorders, applied mainly to lumbar and cervical pain. It is based on the individualized assessment of the patient by observing posture, range of motion, and the pain response to repeated movements; one of its key features is the phenomenon of “centralization”, where pain moves from the limbs toward the spine, indicating an improvement in the patient’s condition [6]. The relevance of MDT in chronic neck pain lies in its capacity to integrate mechanical assessment with patient-centred symptom modulation and active self-management strategies. Although MDT is frequently applied in clinical practice, its effectiveness in chronic neck pain remains debated, with heterogeneous findings across trials and inconsistent methodological quality [7,8]. MDT has gained clinical relevance, especially in patients with directional preference, whose symptoms lessen and centralize or function improves when performing unidirectional spinal movements such as isolated flexion or extension [9]. Through systematic mechanical evaluation and individualized exercise prescription, MDT aims to correct postural deviations and impaired motor control patterns associated with neck pain, reduce pain-related fear, improve movement quality, and enhance self-efficacy, which are key elements in the management of chronic musculoskeletal conditions [10]. Various studies have shown that MDT, compared to conventional interventions or general exercise, can lead to significant improvements in pain, functionality, and emotional well-being in patients with neck pain [11,12,13,14]. Although there is some literature supporting its positive short-term effects, evidence beyond the short term remains inconsistent, and superiority over other active treatments has not been conclusively established. Furthermore, the emphasis on patient education and self-treatment aligns with contemporary biopsychosocial models of pain, making MDT particularly suitable for chronic presentations where behavioural and cognitive components play a central role. Thus, psychosocial factors such as catastrophizing, kinesiophobia, and dysfunctional beliefs are associated with worse clinical outcomes and greater disability [15]. In this context, MDT shows limited effectiveness if it is not accompanied by cognitive–behavioural and psychoeducational approaches [15].

Another treatment strategy for this population is the application of Transcutaneous Electrical Nerve Stimulation (TENS). This is a simple, non-invasive, and low-cost intervention aimed at producing analgesia [16,17], and can be achieved by modulating nociceptive transmission at the dorsal horn of the spinal cord through peripheral electrical stimulation of large sensory afferent fibres—a mechanism known as the “gate control theory” described by Melzack and Wall [18]. Electrical stimulation can activate large-diameter, myelinated A-beta fibres, which have a low threshold for electrical excitation. This activation inhibits pain signals transmitted through smaller-diameter nociceptive afferent fibres, thus reducing the propagation of painful impulses to higher levels of the central nervous system and ultimately decreasing the perception of pain gradually, with effects that can persist after the application [16]. The combination of TENS with other therapeutic interventions has shown results in reducing pain and improving disability in patients with chronic neck pain, especially in the short term [19,20,21]. Evidence suggests that its adjunctive use with other techniques, such as therapeutic exercise, may enhance its benefits, including improvements in pain, cervical muscle strength, and perceived stress. It has even been observed that a single intensive session of TENS could be more helpful in mild cases than in those with more severe symptoms [21]. However, the available evidence is of very low quality, making it difficult to determine the true effectiveness of TENS in this context. Additionally, its effectiveness remains controversial, particularly regarding its possible placebo effect, researchers often fail to specify the stimulation parameters used and employ different equipment configurations, and electrode placement varies greatly among studies [17].

Placebo-controlled trials in exercise-based physiotherapy interventions remain scarce due to the inherent difficulties in designing credible sham procedures, particularly for active approaches such as MDT. Although we were not able to find any placebo-controlled trials specifically in neck pain, there is one RCT evaluating its effects in nonspecific chronic low-back pain that shows a small decrease in pain intensity compared to sham treatment immediately after a 5-week protocol [22]. On the other hand, placebo-controlled trials have demonstrated the analgesic efficacy of electrotherapy modalities in musculoskeletal neck pain, including double-blind randomized studies showing superior outcomes of active biophysical agents compared with sham stimulation [23].

Despite the extended use of exercise, electrotherapy modalities, and MDT in the management of chronic nonspecific neck pain, substantial uncertainty persists regarding their relative effectiveness. Clinically, it remains unclear whether electrotherapy provides additional benefit when combined with exercise therapy and whether MDT offers superior outcomes compared to other active interventions. Given the heterogeneity of existing evidence, the methodological limitations of individual trials, and the lack of clear comparative data, the main objective of this systematic review and meta-analysis is to evaluate the effectiveness of exercise therapy, combined or not with electrotherapy, versus the isolated McKenzie method in reducing disability and pain intensity in adult patients with nonspecific chronic neck pain.

## 2. Materials and Methods

### 2.1. Study Design

This secondary data analysis was conducted through a systematic review including meta-analysis. For its development and reporting, the guidelines and checklist established by the Preferred Reporting Items for Systematic Reviews and Meta-Analyses (PRISMA) [24] were followed; the completed PRISMA checklist is provided as Supplementary Material (Table S1). The study protocol was prospectively registered in the PROSPERO International Prospective Register of Systematic Reviews (Registration ID: CRD420251239356). All methodological procedures followed the recommendations outlined in the Cochrane Handbook for Systematic Reviews of Interventions [25].

### 2.2. Hypothesis

We hypothesize that exercise combined with electrotherapy is more effective in all study variables in the short term compared to exercise applied as a single intervention. Due to the variability in the duration of interventions used across the different studies analyzed, the immediate effects of the interventions (evaluated during or immediately after therapy) are combined or confounded with the sustainability effects in the short, medium, and long term (evaluated after a specific period following the completion of exercise interventions). Considering the previously highlighted research gap, the research questions of this systematic review with meta-analysis are: (1) Does therapeutic exercise combined with electrotherapy, or isolated McKenzie exercises, lead to a sustainable improvement in pain intensity and disability in patients with nonspecific chronic neck pain compared to a control group performing isolated exercise in the short term? (2) Which intervention shows better results in this population?

### 2.3. Inclusion and Exclusion Criteria

The criteria for including or excluding studies were established following the PICO framework (Population, Intervention, Comparator/Control, Outcome). Table 1 details these criteria for both participants and the studies analyzed. The inclusion criteria were: articles published up to 2025, RCTs, studies involving participants aged over 18 years, and articles whose population consisted of adults with nonspecific chronic neck pain. The exclusion criteria were: articles with a PEDro scale score lower than 6; articles involving interventions other than physical activity or electrotherapy interventions; articles whose intervention consisted solely of passive treatments; articles including patients with acute or subacute neck pain; and study protocols.

### 2.4. Search Strategy

The methodology used for this systematic review was based on a bibliographic search in the following databases: PubMed (Medline), Web of Science, and Scopus in June 2025. Searches for potentially relevant articles were conducted using the following Boolean syntax:

((“neck pain” [mesh] OR “cervical pain”) AND ((exercise[mesh] OR “therapeutic exercise”) AND (electrotherapy[mesh] OR “transcutaneous electrical nerve stimulation” OR TENS))) OR ((“neck pain” [mesh] OR “cervical pain”) AND (McKenzie OR “Mechanical Diagnosis and Therapy” OR MDT)).

The articles retrieved from each database through this keyword search strategy were imported into the reference manager Mendeley for subsequent review and removal of duplicates. The article selection process was carried out using a similar search strategy across all databases. Identified studies were screened for eligibility by reviewing titles and abstracts. The remaining full-text articles were examined to verify if they met the inclusion criteria and did not present exclusion reasons. After retrieving the studies, additional articles were identified through manual searching of the reference lists of the selected articles (reference cross-checking). Two independent reviewers participated in the study evaluation process. In case of disagreement between reviewers, it was first discussed, attempting to reach a consensus; if disagreement persisted, a third independent reviewer was consulted.

### 2.5. Data Extraction

The selected studies were examined using common effect size estimators for the disability and pain intensity variables. Data were collected to assess short-term sustainability effects (≤3 months). Relevant information such as descriptive data, PICO, intervention characteristics, study quality, and risk of bias (RoB) was extracted directly from each study. Effect size estimators (pain intensity and disability) were mainly calculated using the Visual Analog Scale (VAS) and the Numeric Pain Rating Scale (NPRS), both ranging from 0 to 10, where the lowest score indicates no pain and the highest score represents the worst imaginable pain. Various studies have demonstrated the equivalence and concurrent validity between the VAS and NPRS [26], allowing their interchangeable use in quantifying cervical pain intensity. Similarly, the calculation of standardized mean differences does not depend on the specific scale used. For the disability variable, the Neck Disability Index (NDI) and the Copenhagen Neck Functional Disability Scale (CNFDS) were used, both showing a high correlation and adequate sensitivity for inclusion in a meta-analysis [27].

### 2.6. Quality of Evidence Assessment

The methodological quality of all included RCTs was assessed using the Physiotherapy Evidence Database (PEDro) [28] scale. Eleven items were evaluated, each scored as 1 if the criterion was met or 0 if it was not. The items are as follows: (1) eligibility criteria; (2) random allocation; (3) concealed allocation; (4) baseline comparability; (5) blinding of subjects; (6) blinding of therapists; (7) blinding of assessors; (8) adequate follow-up; (9) intention-to-treat analysis; (10) between-group comparisons; (11) point estimates and variability.

This scale uses item 1 to establish external validity, items 2 to 9 to assess internal validity, and items 10 and 11 to evaluate the interpretability of the results. Item 1 was not included in the final score, making the maximum possible score on the PEDro scale 10 points. The quality of studies was classified into three categories: high quality (≥6), moderate quality (4–5), and low quality (<4). The PEDro score threshold of ≥6 was selected in accordance with established methodological recommendations and previous systematic reviews in physiotherapy and musculoskeletal research, where scores ≥6 show a reduced RoB [29].

### 2.7. Risk-of-Bias Assessment

The risk of bias of the included studies was independently assessed using the tool developed by the Cochrane Collaboration [30]. Following Cochrane recommendations, bias was rated as outcome-specific rather than study-specific [25]. Results were categorized by the following domains: random sequence generation, allocation concealment, blinding (of researchers, assessors, or participants and outcome assessment), incomplete outcome data, and selective outcome reporting. Each domain was rated as having “low risk”, “high risk”, or “unclear risk” of bias. The final judgments on risk of bias were presented in an aggregated format by study and outcome.

### 2.8. Measures of Treatment Effects

For the primary analysis of treatment effects, Review Manager 5.3 software (RevMan, version 5.3, Copenhagen, Denmark: The Nordic Cochrane Centre, The Cochrane Collaboration, 2014) was used. The quantitative pooling of data in this meta-analysis was performed using standardized mean differences (SMDs) along with the corresponding sample sizes. A random-effects model was selected for continuous outcomes, and 95% confidence intervals were calculated to describe the variance. The results were visually represented using forest plots. The analysis was divided into two main comparisons to address the research questions posed: 1. Therapeutic exercise combined with electrotherapy versus therapeutic exercise alone and 2. McKenzie method exercise versus other interventions, assessing in both cases the variables of pain intensity and disability. In studies with multiple experimental groups, the arm whose intervention showed greater homogeneity and methodological consistency with the objectives of this review was included. In studies featuring more than one relevant intervention group, both groups were incorporated into the meta-analysis sharing the same control group; in such cases, the sample size of the control group was divided among the relevant comparisons, following the recommendations of the Cochrane Handbook for Systematic Reviews of Interventions [25]. For meta-analysis purposes only, when comparing the McKenzie method with other therapies, the group performing McKenzie exercises alone was considered the experimental group. Due to differences in follow-up periods across the included studies, it was decided to analyze short-term outcomes using the data collected immediately after the intervention ended. The magnitude of effect was interpreted as follows: small (SMD: 0–0.2), moderate (SMD: 0.2–0.5), large (SMD: 0.5–0.8), or very large (SMD > 0.8). The clinical heterogeneity of the results was assessed using the I^2^ statistic, with I^2^ > 50% considered substantial heterogeneity. This heterogeneity was considered when determining the overall certainty of the evidence, applying the Grading of Recommendations Assessment, Development and Evaluation (GRADE) [31] methodology. Factors that could lower the certainty included: RoB, inconsistency of results, lack of direct applicability (no generalizability), imprecision due to data dispersion, and reporting bias. Finally, the quality of the evidence was categorized as very low, low, moderate, or high.

## 3. Results

### 3.1. Data Collection

**Figure 1:**
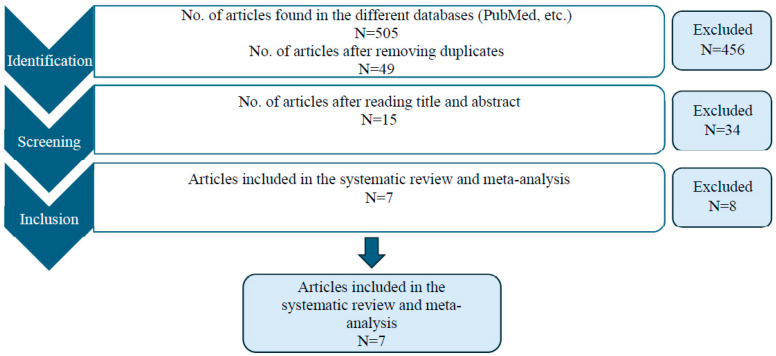
Search, selection, and synthesis of the included studies.

Data collection from the databases was completed in June 2025. Figure 1 illustrates the search process, as well as the stages of study selection and inclusion.

### 3.2. Characteristics of the Studies and Interventions

A total of seven studies were included in the qualitative and quantitative analyses [32,33,34,35,36,37,38]. Their characteristics and baseline results are presented in Table 2. For each selected study, methodological aspects, participant characteristics, and the most relevant findings are described. Overall, the analyzed sample comprised 441 individuals with nonspecific chronic neck pain. Of the seven included RCTs, five used single blinding, while two applied double blinding [32,36]. Regarding the interventions, three studies had three intervention arms [32,33,34] and the remaining studies had two arms. The main inclusion criterion was chronic neck pain lasting ≥3 months without specific etiology. All studies included both male and female participants in similar proportions. Pain outcomes were assessed using the Visual Analogue Scale (VAS) in three studies [33,34,37] and the Numeric Pain Rating Scale (NPRS) in the others, while the primary measure of disability was the Neck Disability Index (NDI); only one study used the Copenhagen Neck Functional Disability Scale (CNFDS) [34].

There was variability in the timing of post-intervention assessment: the shortest studies assessed outcomes at 2 weeks [37,38] or 4 weeks [32], while the most common evaluation point was at 6 weeks [33,34,35,36]. For the purposes of this meta-analysis and in line with this study’s inclusion criteria, we chose to exclude the group designated as the control in the paper by Abdel-Aziem et al. [34]. as it involved only a passive intervention; instead, the control group was replaced by the group that performed deep neck flexor exercises (DNF). The specific characteristics of each intervention are detailed in Table 3. The frequency of application in the studies that combined exercise with electrotherapy ranged from 2 to 5 sessions per week, with a total of between 8 and 24 sessions; in contrast, the McKenzie studies applied treatment daily, 3 days per week, or 5 days per week, totalling between 18 and 42 sessions. There was no clear consensus regarding the electrotherapy parameters to be used; similarly, exercise dosage was highly heterogeneous across all studies; even among those that, technically, applied the same or similar types of exercise, comparable dosages were not used. The type of electrotherapy used in combination with exercise included high-frequency TENS (80–100 Hz) in two intervention groups, low-frequency TENS (4 Hz) in one group, and IFC (interferential current) in three intervention groups. Meanwhile, the isolated McKenzie exercises were compared to other exercise programmes, such as neck stability exercises (NSEs), cranio-cervical flexion exercises (CCF), and deep neck flexor exercises (DNF).

### 3.3. Quality and Risk of Bias of the Included Studies

The assessment of methodological quality of the studies is presented in Table 4. Overall, study quality scores ranged from 6 to 10 points out of a maximum of 10, with an average methodological quality score across the included studies of 7.86/10, reflecting good methodological quality. The lowest score obtained on the PEDro scale was 6 in the study by Abdel-Aziem et al. [34]., which is still considered good quality. The highest score was reported by Martins-de-Sousa et al. [32]., achieving the maximum score. The most common score among the studies was 8/10. The items with the lowest scores were: (1) related to therapist blinding, which was only fulfilled by Martins-de-Sousa et al. [32] and (2) related to participant blinding, which was only fulfilled by two of the seven included studies.

**Figure 2:**
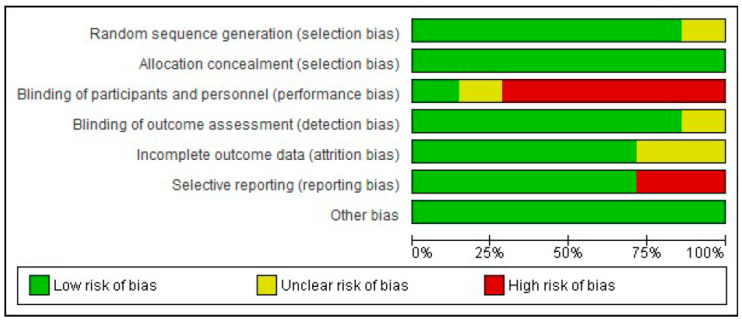
Risk of bias. Risk of bias summary: review authors’ judgements about each risk of bias item for each included study.

**Figure 3:**
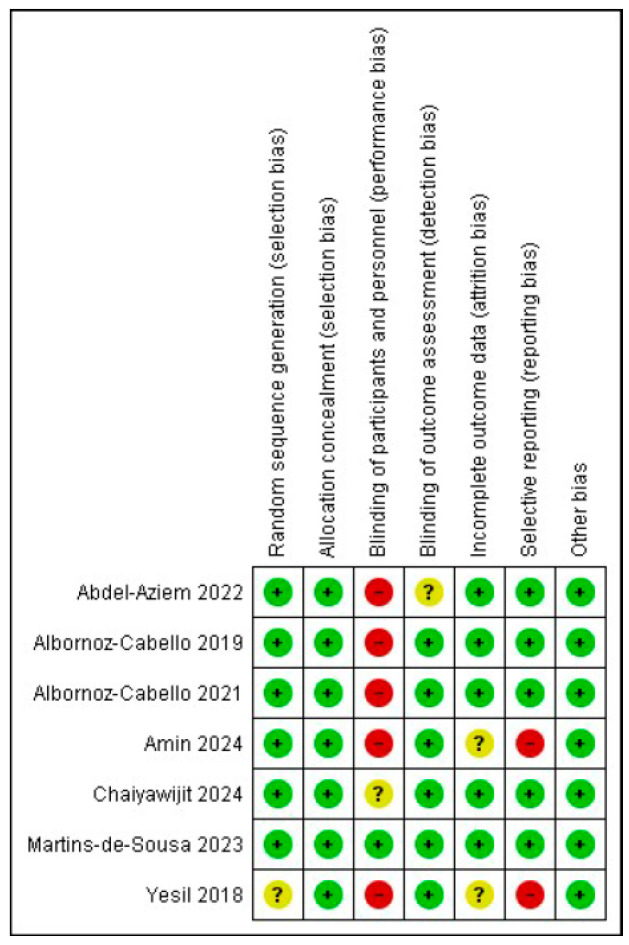
Risk of bias. Risk of bias graph: review authors’ judgements about each risk of bias item presented as percentages across all included studies. Green “+” means low risk; yellow “?” means unclear risk/not enough information; red “-“ means high risk.

Risk-of-bias assessment of the included studies is presented in Figure 2 and Figure 3. More than 80% of the included studies adequately fulfilled key items such as random sequence generation, allocation concealment and blinding of outcome assessment. In most of the included studies, blinding of participants and personnel was not feasible due to the inherent nature of the physiotherapeutic interventions evaluated. This limitation, which is common in this type of trials in the field of physiotherapy, especially when active treatments are applied, entails a risk of performance bias, mainly when subjective outcomes such as pain or disability are assessed.

Nevertheless, since this bias similarly affects all comparisons and has been well documented in previous studies, it was contemplated when interpreting the results and assessing the certainty of the evidence. Therefore, performance bias was considered potentially relevant only in those studies that, in addition to lacking blinding of participants and personnel, also presented high or unclear risk of bias in blinding of outcome assessors, as the latter can mitigate the impact of performance bias. Four studies were considered to have a low risk of bias [32,36,37,38], only one study was rated as unclear risk due to possible performance bias [34], and high risk of attrition and reporting bias was observed in two trials [33,35] for not providing complete data on the disability outcome.

Complete information about the pain and disability outcomes of each study is shown in Table 5.

### 3.4. Therapeutic Exercise (TE) + Electrotherapy vs. TE Alone

**Figure 4:**
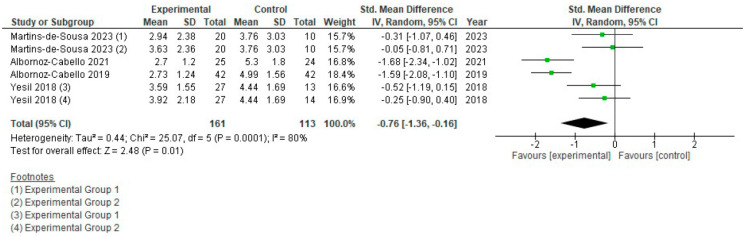
Forest plot of comparison: pain intensity (T.E. + electrotherapy vs. T.E.).

Pain intensity: Six groups from four different studies were included, comprising a total of 274 participants, Figure 4. The standardized mean difference (SMD) was −0.76 [−1.36, −0.16] (*p* = 0.01; 95% CI), suggesting a large effect size in favour of the experimental group. There was very high heterogeneity (I^2^ = 80%), indicating substantial variability among studies, possibly explained by the different outcome measures used (VAS or NPRS) and the specific characteristics of each intervention. The overall effect consistently favoured pain reduction in studies applying TE + electrotherapy, with no results in the opposite direction; therefore, the certainty of the evidence was considered moderate.

**Figure 5:**
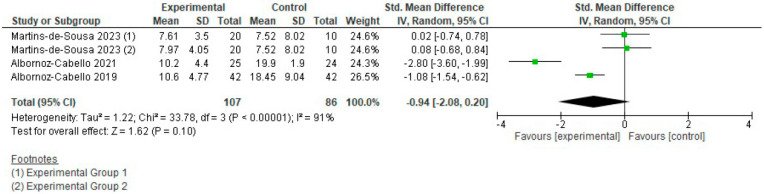
Forest plot of comparison: disability (T.E. + electrotherapy vs. T.E.).

Disability: Four groups from three different studies were included, with a total of 193 subjects, Figure 5. The study by Yesil et al. [33]. could not be included in the meta-analysis for this variable due to incomplete reporting of results. The SMD was −0.94 [−2.08, 0.20] (*p* = 0.1; 95% CI), suggesting a very large effect size supporting the experimental group, which was not statistically significant (*p* = 0.1) as the confidence interval (CI) crossed the line of no effect and the heterogeneity was extremely high (I^2^ = 91%). Therefore, we cannot establish solid differences in disability levels backing either group, and the certainty of the evidence is considered low.

**Figure 6:**
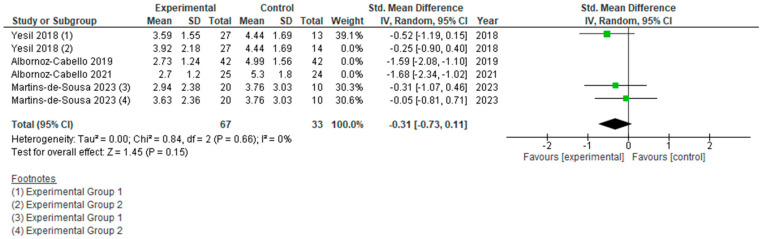
Forest plot of comparison: pain intensity (T.E. + TENS vs T.E.).

**Figure 7:**
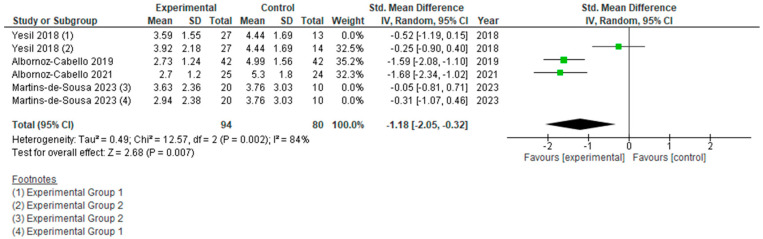
Forest plot of comparison: pain intensity (T.E. + IFC vs T.E.).

**Figure 8:**
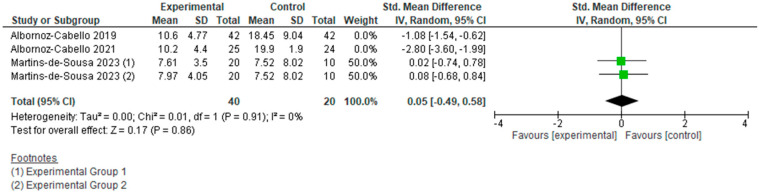
Forest plot of comparison: disability (T.E. + TENS vs T.E.).

**Figure 9:**
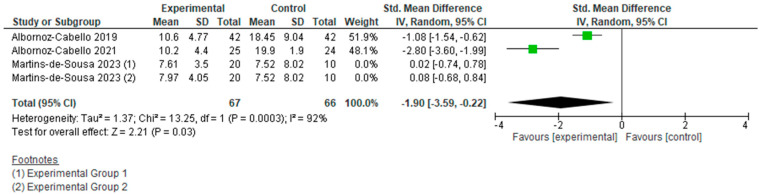
Forest plot of comparison: disability (T.E. + IFC vs T.E.).

However, if we analyze the two types of currents used separately (Figure 6, Figure 7, Figure 8 and Figure 9), we can see how each actually affects the results of this meta-analysis. We found that when TENS + TE was applied, the results were not statistically significant either in pain reduction (SMD −0.31 [−0.73, 0.11]; (*p* = 0.15; 95% CI)) or in disability levels (SMD 0.05 [−0.49, 0.58]; (*p* = 0.86; 95% CI)). In contrast, when IFC + TE was applied, both variables improved with strong significance and a very large effect size: for pain intensity, SMD −1.18 [−2.05, −0.32]; (*p* = 0.007; 95% CI) and for disability level, SMD −1.90 [−3.59, −0.22]; (*p* = 0.03; 95% CI). Therefore, no benefits were found when adding TENS to an exercise programme; on the contrary, the results after applying IFC combined with exercise were much better in terms of pain reduction and improvement in disability than those obtained after exercise alone.

### 3.5. McKenzie vs. Other Therapies

**Figure 10:**
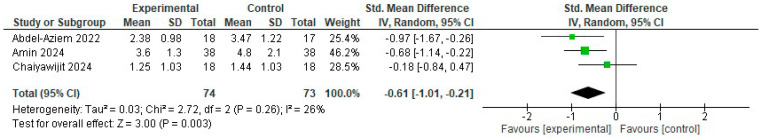
Forest plot of comparison: pain intensity (McKenzie vs. control).

Pain intensity: Three groups from three different studies were included, with a total of 147 subjects, Figure 10. The SMD was −0.61 [−1.01, −0.21] (*p* = 0.003; 95% CI), assuming a large effect size in favour of the experimental group, with statistically significant differences (*p* = 0.003). The CI clearly lies on the experimental group side, and the inconsistency of the results was quite low (I^2^ = 26%); therefore, the certainty of the evidence was initially considered high. However, due to the RoB in the included studies, the level of evidence was downgraded to moderate. These findings show that the groups applying isolated McKenzie exercises achieved a significant reduction in pain compared to the control group.

**Figure 11:**
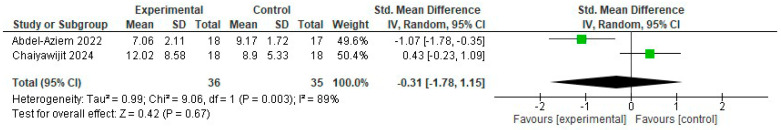
Forest plot of comparison: disability (McKenzie vs. control).

Disability: Two groups from two different studies were included, with a total of 71 participants, Figure 11. The study by Amin et al. [35] was excluded from this analysis because it did not report the respective results. The SMD was −0.31 [−1.78, 1.15] (*p* = 0.67; 95% CI), resulting in a moderate effect size and a CI that clearly crosses the line of no effect. The heterogeneity of this analysis was very high (I^2^ = 89%). Although the overall effect supports the experimental group, it is not statistically significant (*p* = 0.67), so neither group was shown to be superior for this outcome. The certainty of the evidence was initially considered moderate, but due to the RoB in the included studies, the level of evidence was downgraded to low.

## 4. Discussion

Regarding therapeutic exercise combined with electrical stimulation, the results showed a statistically significant reduction in pain (SMD −0.76 [−1.36, −0.16] (*p* = 0.01; 95% CI)), but not in disability (SMD −0.94 [−2.08, 0.20] (*p* = 0.1; 95% CI)), compared to exercise alone. The effect size for the “pain” variable was large. Nevertheless, high inconsistency substantially limits the confidence in the clinical relevance of this effect. However, these results are largely influenced by the effect of applying IFC, since the use of TENS did not lead to additional benefits over exercise in either variable, whereas IFC achieved significant improvements both in pain (SMD −1.18 [−2.05, −0.32] (*p* = 0.007; 95% CI)) and disability (SMD −1.90 [−3.59, −0.22] (*p* = 0.03; 95% CI)), with a very large effect size. This apparent superiority of IFC over TENS should be interpreted cautiously, as it is based on a small number of heterogeneous studies with limited sample sizes and short-term follow-up, which fundamentally constrain the strength of inference.

Considerable heterogeneity was identified in both analyses (I^2^ > 80%). This heterogeneity is possibly determined by the variability in electrical stimulation parameters, frequency and duration of treatments; the demographic characteristics of the patients included; the type of exercise performed; treatment progression; or other individual factors. Such variability is inherent to complex non-pharmacological rehabilitation interventions, which are commonly tailored to individual patient needs, symptom presentations, and clinical context, reflecting real-world physiotherapy practice. Accordingly, the present findings do not support definitive clinical recommendations but rather highlight current evidence gaps and the need for more standardized and methodologically robust trials. Further research into these two interventions separately, using well-defined parameters, is encouraged to build higher-quality evidence.

The isolated McKenzie method achieved better results for reducing pain intensity than the other therapies used as comparators, with an SMD of −0.61 [−1.01, −0.21] (*p* = 0.003; 95% CI) and low inconsistency (I^2^ = 26%), which gives robustness and reliability to the results. On the other hand, no superior benefits of McKenzie exercises over other therapies were found in reducing disability (SMD −0.31 [−1.78, 1.15] (*p* = 0.67; 95% CI)). However, interpretation of these findings must consider that included studies did not adequately report key components of the MDT assessment process, such as directional preference classification and individualized exercise prescription.

Overall, the evidence suggests that exercise combined with electrotherapy may be more effective than exercise alone in reducing pain; nonetheless, these must be regarded as preliminary findings, given the substantial heterogeneity, methodological limitations, and short-term follow-up of the included studies. However, the real benefits of electrotherapy appear to lie with IFC, which achieved clear improvements in both pain and disability, whereas TENS seems to provide no additional benefits over therapeutic exercise. These findings should be interpreted as a hypothesis rather than a robust statement. Alternatively, the evidence appears to support the superiority of the isolated McKenzie method in improving pain compared to other therapies, though it was not superior in the disability outcome.

The nature of the included studies and the way data were reported made it difficult to synthesize firm conclusions. Most studies only collected data immediately after the intervention, limiting consideration of the results to the short term. The lack of consensus on the parameters used for therapeutic currents complicates comparison and increases inconsistency in the analysis. Additionally, there seems to be no clear criterion for exercise dosage in studies of active therapies, nor any apparent logic or methodology for setting the number of sets, repetitions, and rest periods, making the literature difficult to compare even when the exercise programme applied is, theoretically, similar. This lack of conceptual and methodological standardization in exercise prescription represents a major limitation, as exercise is the cornerstone of contemporary neck pain management and cannot be considered as a homogeneous intervention. These methodological limitations highlight the need for future trials employing standardized, well-defined intervention protocols and longer follow-up periods.

The findings of this meta-analysis are consistent with previous studies [17] suggesting that TENS has limited effects on pain intensity in patients with chronic neck pain, although more high-quality scientific contributions are still needed to draw robust conclusions. In line with the results of this research, Rampazo et al. [19] also observed statistically significant improvements in pain reduction when electrotherapy was used as an adjunct treatment to other interventions; however, they did not individually study the specific effect of each electrotherapy modality. In contrast, the present review attempted to isolate the effects of individual modalities, but the resulting evidence remains limited by small sample sizes and substantial heterogeneity. We did find discrepancies regarding disability levels, where they did report favourable results. Although the study populations were similar, one of the determining factors in the results of this meta-analysis may have been the chronicity of the pain. However, it is essential to acknowledge that, in addition to the limited evidence available on the application of electrotherapy in subjects with these characteristics, many studies do not adequately report the technical parameters of the intervention; the methodological quality of the studies is, in many cases, low; and the long-term effects remain uncertain, as previously reported in other meta-analyses [39]. Regarding the McKenzie method, there are currently no prior meta-analyses on cervical pathology at the time of publication of this study. However, there are studies investigating its use in patients with low back pain, although their results remain controversial and appear to depend on which intervention it is compared against [9,40,41].

From a clinical point of view, these findings have important implications. Rather than supporting specific modality-based recommendations, the present results reinforce the central role of active therapies as the core intervention for chronic nonspecific neck pain, while suggesting that electrotherapy modalities may offer, at best, short-term adjunctive analgesic effects that might contribute to early symptom management. Furthermore, the results question the exclusive and prioritized use of the McKenzie method. While it is effective in reducing pain in the short term, it does not appear to offer superior benefits over other therapies in terms of disability levels. Its clinical value may therefore depend more on appropriate patient selection, accurate classification, and multimodal rehabilitation programmes rather than on its isolated application. These findings reinforce the need for a complex treatment model that combines active therapeutic exercise with educational interventions and, when indicated, low-risk analgesic strategies such as electrostimulation.

The included studies present several limitations that affect the interpretation of the results. The most notable is the high clinical and methodological heterogeneity, for example, differences in treatment duration, number of sessions, intensity, electrostimulation parameters, and exercise dosage. The risk of bias in the blinding of participants and therapists was particularly high; although this is common in physiotherapy studies due to the nature of the interventions and, at times, the impossibility of blinding, it limits the internal validity of the results. Some studies did not clearly report exercise dosage parameters, which compromises clinical replicability. Another important aspect is that the studies applying the McKenzie method did not report the specific assessment on which their treatments were based; therefore, the exercises selected might not have been the most beneficial for the participants, contradicting the fundamental premises of the method and potentially biassing its effectiveness. Finally, most studies focused on short-term outcomes without evaluating sustained effects in the medium or long term, which limits the generalizability of the findings.

Within the scope of this review, there are also limitations that should be acknowledged. Firstly, although rigorous inclusion criteria were applied, the limited number of available studies for each comparison reduced the statistical power of the analyses and the possibility of conducting more detailed subgroup analyses. It was also not possible to evaluate the differential effect of home exercise or physiotherapist supervision on the outcomes, a highly relevant factor in real clinical practice. The high statistical heterogeneity is another limiting factor, as it lowers the certainty of the estimated effects. Although a random-effects model was used to mitigate this variability, methodological inconsistency remains a potential source of bias. Attempts were made to contact the authors of studies with incomplete data, but no response was obtained. As a result, relevant information was lost, particularly regarding the disability variable, which had reduced representation in the meta-analysis.

## 5. Conclusions

Electrotherapy may provide short-term analgesic effects when used as an adjunct to exercise in adults with nonspecific chronic neck pain. However, due to substantial methodological limitations, high heterogeneity, small sample sizes, and the short-term nature of the available evidence, these findings should be interpreted with considerable caution and cannot support firm clinical recommendations. Particularly, although exercise combined with interferential current demonstrated greater short-term improvements in pain and disability than exercise alone, this apparent advantage should not be taken as an absolute certainty.

Although isolated McKenzie exercises achieved superior short-term pain reduction compared to other interventions, they did not produce significant improvements in reducing short-term disability in patients with nonspecific chronic neck pain.

The present findings highlight important evidence gaps and underline the need for high-quality randomized controlled trials with standardized intervention protocols, adequate reporting, and medium- to long-term follow-up to better establish the comparative effectiveness of exercise-based interventions and electrotherapy.

## Figures and Tables

**Table 1:** Inclusion and exclusion criteria for studies and participants.

Criterion	Inclusion	Exclusion
Study design	Randomized controlled trial (RCT).	Case studies. Case–control studies. Cohort studies. Systematic reviews.
Population	Adults with chronic nonspecific neck pain (chronic ≥ 3 months at the time of study inclusion).	Children or adolescents < 18 years old. Patients with acute/subacute neck pain. Patients with cervical tumours, mechanical causes, neuritis, fractures, etc.
Intervention	Exercises based on the McKenzie method/therapeutic exercise/specific cervical extensor exercises/stabilization exercises/cranio-cervical flexion exercises (CCF or NSE)/deep cervical flexor (DNF) exercises/scapulothoracic exercises. Electrical stimulation: TENS/interferential currents (IFCs).	Any intervention differing from those mentioned under inclusion.
Control/comparator	Active.	Passive.
Outcome	Pain measurement (VAS, NSR, NPRS) and disability (NDI, CNFDS).	
Other	Publication date: up to 2025. Full-text availability. PEDro scale score ≥ 6 (high quality).	

**Table 2:** Characteristics of the included studies.

Author	Year	Study Design/Arms	Inclusion Criteria	*N* Total	Mean Age (Years)	Sex (F/M)	Baseline Pain (Scale)	Baseline Disability	Measurement Timepoints
Martins-de-Sousa [32]	2023		Chronic neck pain. Sedentary patients.				NPRS (0–10)	NDI	Baseline, week 4, 4 weeks follow-up.
		EG_1_		20	29.35 ± 8.8	15/5	5.33 ± 2.44	11.27 ± 2.46	
		EG_2_		20	31.55 ± 6.13	14/6	5.00 ± 2.30	11.73 ± 5.05	
		CtrlG		20	30.40 ± 7.74	12/8	5.41 ± 2.85	11.11 ± 8.90	
Albornoz-Cabello [38]	2021		Chronic neck pain, NSNP grade I or II.				NPRS (0–10)	NDI	Baseline, week 2.
		EG		25	49.32 ± 8.17	15/10	6.62 ± 1.10	24.92 ± 8.39	
		CtrlG		24	44.50 ± 12.97	20/4	6.62 ± 1.42	27.96 ± 9.53	
Albornoz-Cabello [37]	2019		Medical diagnosis of chronic neck pain				VAS (0–10)	NDI	Baseline, week 2.
		EG		42	49.81 ± 9.52	29/13	6.60 ± 1.30	26.45 ± 7.65	
		CtrlG		42	44.52 ± 11.77	33/29	6.23 ± 1.49	26.10 ± 9.68	
Yesil [33]	2018		Chronic neck pain				VAS (0–10)	NDI	Baseline, week 6, 12 weeks follow-up.
		EG_1_		27	38.59 ± 9.19	19/8	6.59 ± 2.09	42.07 ± 17.65	
		EG_2_		27	39.74 ± 8.76	21/6	5.96 ± 1.12	40.07 ± 14.43	
		CtrlG		27	36.03 ± 7.86	16/11	6.88 ± 1.60	45.03 ± 18.46	
Amin [35]	2024	EG CtrlG	Nonspecific chronic neck pain	38	40.11 ± 7.00	14/24	NPRS (0–10)	NDI	Baseline, week 6.
							5.90 ± 1.00	NR	
				38	42.05 ± 8.93	20/18	5.80 ± 1.30	NR	
Chaiyawijit [36]	2024	EG CtrlG	Chronic neck pain	18 18	37.67 ± 8.62 36.94 ± 8.45	11/7 10/8	NPRS (0–10) 5.36 ± 1.73 5.72 ± 1.63	NDI 30.23 ± 10.05 36.51 ± 13.04	Baseline, week 6 (pain measured weekly)
Abdel-Aziem [34]	2022		Nonspecific chronic neck pain				VAS (0–10)	CNFDS	Baseline, week 6.
		EG_1_		18	43.06 ± 8.68	7/11	6.28 ± 1.79	15.39 ± 2.36	
		EG_2_		17	41.24 ± 7.82	7/10	6.47 ± 1.84	15.76 ± 3.99	
		CtrlG		20	41.60 ± 8.99	8/12	6.15 ± 1.08	14.75 ± 3.86	

For each included study, methodological aspects and participant characteristics are shown. Values are expressed as mean ± standard deviation. EG: experimental group; CtrlG: control group; f: female; m: male; VAS: Visual Analogue Scale; NPRS: Numeric Pain Rating Scale; NDI: Neck Disability Index; CNFDS: Copenhagen Neck Functional Disability Scale. Assessments that were included in the study protocol, but whose exact values could not be found in the manuscript, are marked as not reported values (NR).

**Table 3:** Individual characteristics of each study.

Author and Year	Intervention	Duration	Frequency	Session Duration	Electrotherapy Parameters	Exercises	Volume (Sets × Repetitions)	Rest Between Sets
Martins-de-Sousa 2023 [32]		4 weeks	2 sessions/week (8 sessions)	60 min: 30 min TENS + 30 min ET	Rectangular biphasic symmetric current. Intensity adjusted every 5 min to tolerance threshold.	ET: 6 exercises (1 active ROM + 1 neurodynamics + 4 isometric exercises)	3 × 3 active ROM 3 × 1 min neurodynamics 3 × 10 s isometrics	2 min
	EG_1_: ET + high-frequency TENS				100 Hz, 100 µs			
	EG_2_: ET + low-frequency TENS				4 Hz, 100 µs			
	CtrlG: ET + placebo TENS				0 mA			
Albornoz-Cabello 2021 [38]		2 weeks	5 sessions/week (10 sessions)	25–45 min ET + 25 min IFC.	Bipolar interferential current. Intensity according to tolerance.	ET: bilateral active stretching + pain education (sessions 1 and 2). Isometric and eccentric exercises combined with oculomotor training (from session 3 onwards).	3 × 3–5 repetitions, each 3–10 s	NR
	EG: ET + IFC				Carrier frequency 4000 Hz, modulated frequency 60 Hz, sweep 90 Hz			
	CtrlG: ET				NA			
Albornoz-Cabello 2019 [37]		2 weeks	5 sessions/week (10 sessions)	25–45 min ET + 25 min IFC + 30–45 min HE.	Bipolar interferential current. Intensity according to tolerance.	ET: postural education + bilateral active stretching (sessions 1 and 2) + isometric exercises (from session 3) + oculomotor training (from session 4). HE: repeat ET at home once daily.	3 × 3–5 stretching 3–10 s isometrics 5–10 s	NR
	EG: supervised ET + IFC				Carrier frequency 4000 Hz, modulated frequency 60 Hz, sweep 90 Hz			
	CtrlG: supervised ET				NA			
Yesil 2018 [33]		6 weeks	5 sessions/week (first 3 weeks) + 3 sessions/week HE (last 3 weeks)	~60 min	TENS or IFC, intensity at sensory threshold.	NSE: postural education, 5 min jogging, cervical, pectoral and scapular stretching, cervical isometric exercises, upper extremity resistance exercises. HE: NSE	2 × 15 (resistance exercises), stretching 1 min, isometrics 10–15 reps x10–15 s	5 min
	EG_1_: TENS + NSE				TENS: 80 Hz, 10–30 mA, 25 min.			
	EG_2_: IFC + NSE				IFC: carrier frequency 4000 Hz, modulated frequency 100 Hz, 25 min			
	CtrlG: NSE				NA			
Amin, 2024 [35]		6 weeks	3 sessions/week (18 sessions) + HE twice/day	30–45 min.	NA	McKenzie method, NSE, HE (repeat assigned exercises at home).		
	EG: McKenzie + NSE				NA	McKenzie method. NSE: cervical isometric exercises + scapular retractions with resistance bands + external rotation and shoulder abduction with resistance bands. HE.	McKenzie: 10–15 reps per side, 30–45 min. NSE: isometrics 10 reps × 10 s, resistance bands 3 × 10	NA
	CtrlG: McKenzie				NA	McKenzie method. HE.	McKenzie: 10–15 reps per side, 30–45 min	NA
Chaiyawijit, 2024 [36]		6 weeks	Daily	NA	NA	Home-based CCF or McKenzie exercises.		
	EG: CCF				NA	Home-based CCF.	10 sets × 10 s (twice daily).	NA
	CtrlG: McKenzie				NA	Home-based McKenzie method.	6 × 10.	NA
Abdel-Aziem, 2022 [34]		6 weeks	5 sessions/week (30 sessions)	>60 min.	PTA: TENS (80 Hz, 10–30 mA, 30 min) + US (continuous, 1 MHz, 1.5 W/cm^2^, 10 min) + IRI (20 min)	Cervical stretching, cervical isometric exercises, scapulothoracic exercises, DNF, McKenzie.		NA
	EG_1_: PTA + DNF				PTA	Cervical stretching, cervical isometric exercises, scapulothoracic exercises, DNF.	Stretching: 3 × 30 s isometrics: 3 × 10 × 6 s Scapular: twice daily DNF: 10 × 10 s	NA
	EG_2_: PTA + Mckenzie				PTA	McKenzie.	5–6 × 10–15	NA
	CtrlG: PTA				PTA	NA	NA	NA

Control group (CtrlG); experimental group (EG); not reported (NR); not applicable (NA); Transcutaneous Electrical Nerve Stimulation (TENS); range of motion (ROM); home exercises (HEs); neck stabilization exercises (NSEs); craniocervical flexion exercises (CCF); physical therapy agents (PTAs); ultrasound therapy (US); infrared irradiation (IRI); deep neck flexor exercises (DNF).

**Table 4:** Methodological quality assessed using the PEDro scale.

Author and Year	Item											Total Score
	1	2	3	4	5	6	7	8	9	10	11	
Amin 2024 [35]	1	1	1	1	0	0	1	1	1	1	1	8/10
Chaiyawijit 2024 [36]	1	1	1	1	1	0	1	1	0	1	1	8/10
Martins-de-Sousa 2023 [32]	1	1	1	1	1	1	1	1	1	1	1	10/10
Abdel-Aziem 2022 [34]	1	1	1	1	0	0	0	1	0	1	1	6/10
Albornoz-Cabello 2021 [38]	1	1	1	1	0	0	1	1	1	1	1	8/10
Albornoz-Cabello 2019 [37]	1	1	1	1	0	0	1	1	1	1	1	8/10
Yesil 2018 [33]	0	1	1	1	0	0	1	1	0	1	1	7/10

Item number 1 is not included in the total score.

**Table 5:** Pain and disability outcomes across the studies.

Author	Group	Pain			Disability		
		Baseline	Post-Treatment	Follow-Up	Baseline	Post-Treatment	Follow-Up
Martins-de-Sousa 2023 [32]	EG_1_	5.33 ± 2.44	2.94 ± 2.38	3.33 ± 2.00	11.27 ± 2.46	7.61 ± 3.50	-
	EG_2_	5.00 ± 2.30	3.63 ± 2.36	3.31 ± 2.21	11. 73 ± 5.05	7.97 ± 4.05	-
	CtrlG	5.41 ± 2.85	3.76 ± 3.03	3.64 ± 3.33	11.11 ± 8.90	7.52 ± 8.02	-
Albornoz-Cabello 2021 [38]	EG	6.62 ± 1.10	2.70 ± 1.20	-	24.92 ± 8.39	10.20 ± 4.40	-
	CtrlG	6.62 ± 1.42	5.30 ± 1.80	-	27.96 ± 9.53	19.90 ± 1.90	-
Albornoz-Cabello 2019 [37]	EG	6.60 ± 1.30	2.73 ± 1.24	-	26.45 ± 7.65	10.60 ± 4.77	-
	CtrlG	6.23 ± 1.49	4.99 ± 1.56	-	26.10 ± 9.68	18.45 ± 9.04	-
Yesil 2018 [33]	EG_1_	6.59 ± 2.09	3.59 ± 1.55	3.00 ± 1.35	42.07 ± 17.65	NR	NR
	EG_2_	5.96 ± 1.12	3.92 ± 2.18	3.40 ± 1.98	40.07 ± 14.43	NR	NR
	CtrlG	6.88 ± 1.60	4.44 ± 1.69	3.65 ± 1.32	45.03 ± 18.46	NR	NR
Amin 2024 [35]	EG	5.90 ± 1.00	4.80 ± 2.10	-	NR	NR	-
	CtrlG	5.80 ± 1.30	3.60 ± 1.30	-	NR	NR	-
Chaiyawijit 2024 [36]	EG	5.36 ± 1.73	1.44 ± 1.03	-	30.23 ± 10.05	8.90 ± 5.33	-
	CtrlG	5.72 ± 1.63	1.25 ± 1.03	-	36.51 ± 13.04	12.02 ± 8.58	-
Abdel-Aziem 2022 [34]	EG_1_	6.28 ± 1.79	3.47 ± 1.22	-	15.39 ± 2.36	9.17 ± 1.72	-
	EG_2_	6.47 ± 1.84	2.38 ± 0.98	-	15.76 ± 3.99	7.06 ± 2.11	-

Values are expressed as mean ± standard deviation. Assessments that were not included in the study design are marked with a dash “-”. Assessments that were included in the study protocol, but whose values could not be found in the manuscript, are marked as not reported values (NR).

## Data Availability

Data is contained within the article. Further inquiries can be directed to the corresponding author.

## Supplementary Materials

The following supporting information can be downloaded at https://www.mdpi.com/article/10.3390/jcm15051689/s1. Table S1: PRISMA checklist.

## Abbreviations

The following abbreviations are used in this manuscript:

NSNP	Nonspecific neck pain
SCMs	Sternocleidomastoid muscles
MDT	Mechanical Diagnosis and Therapy
VAS	Visual Analog Scale
TENS	Transcutaneous Electrical Nerve Stimulation
PRISMA	Preferred Reporting Items for Systematic Reviews and Meta-Analyses
NPRS	Numeric Pain Rating Scale
NDI	Neck Disability Index
CNFDS	Copenhagen Neck Functional Disability Scale
PEDro	Physiotherapy Evidence Database
SMD	Standardized mean difference
GRADE	Grading of Recommendations Assessment, Development and Evaluation
RoB	Risk of bias
NSE	Neck stability exercise
CCF	Cranio-cervical flexion exercises
DNF	Deep neck flexor exercises
